# Plant-growth promoting activity of three fungal endophytes isolated from plants living in dehesas and their effect on *Lolium multiflorum*

**DOI:** 10.1038/s41598-023-34036-8

**Published:** 2023-05-05

**Authors:** C. García-Latorre, S. Rodrigo, Y. Marin-Felix, M. Stadler, O. Santamaria

**Affiliations:** 1 School of Agricultural Engineering, University of Extremadura, Avda. Adolfo Suárez s/n, 06007 Badajoz, Spain; 2 Institute of Dehesa Research (INDEHESA), University of Extremadura, Avda. de Elvas s/n, 06006 Badajoz, Spain; 3 Department of Microbial Drugs, Helmholtz-Centre for Infection Research, Inhoffenstrasse 7, 38124 Braunschweig, Germany; 4 Institute of Microbiology, Technische Universität Braunschweig, Spielmannstraße 7, 38106 Braunschweig, Germany; 5 Department of Plant Production and Forest Resources, Sustainable Forest Management Research Institute (iuFOR), University of Valladolid, Avda. de Madrid 57, 34004 Palencia, Spain

**Keywords:** Plant physiology, Field trials, Agroecology

## Abstract

Endophytic fungi have been demonstrated to produce bioactive secondary metabolites, some of which promote plant growth. Three endophytic fungi isolated from healthy plants living in dehesas of Extremadura (Spain) were identified and evaluated for their ability to produce phytohormone-like substances, antioxidant activity, total polyphenol content, phosphate solubilization ability and siderophore and ammonia production. The filtrates and extracts produced by the three endophytes were applied to *Lolium multiflorum* seeds and seedlings under both in vitro and greenhouse conditions, to analyse their influence on plant growth traits such as germination, vigour index, chlorophyll data, number and length of leaves and roots, and dry weight. All three endophytes, which were identified as *Fusarium avenaceum*, *Sarocladium terricola* and Xylariaceae sp., increased the germination of *L. multiflorum* seeds by more than 70%. Shoot and root length, plant dry weight and the number of roots were positively affected by the application of fungal filtrates and/or extracts, compared with controls. The tentative HPLC–MS identification of phytohormone-like substances, such as gibberellin A2 and zeatin, or the antioxidant acetyl eugenol, may partially explain the mechanisms of *L. multiflorum* plant growth promotion after the application of fungal filtrates and/or extracts.

## Introduction

Plant production in agriculture has traditionally been associated with the use of inorganic fertilizers and synthetic pesticides to improve crop productivity. However, such practices may negatively affect soil quality and favour pollution processes in the moderate and long term^1,2^. This aspect might be particularly relevant in extensive agricultural systems, such as dehesas in Spain (known as *montados* in Portugal), which are extremely vulnerable because of their harsh climatic conditions and poor soils^3^. Dehesas are agrosilvopastoral systems originating from clearing of the tree layer, which is composed primarily of holm oak (*Quercus ilex*) trees, in the traditional Mediterranean forest of the southwestern Iberian Peninsula^4^. These ecosystems have been able to survive because of their multifunctional characteristics, which support a balanced of livestock, agricultural and forestry productivity^5,6^, while providing important ecosystem services^7^. However, the low yield and high interannual variability of herbage^3^ limit the profitability of the farms in these areas, and require livestock feed to be supplemented with external fodder resources. This situation has recently been aggravated by various factors, such as the deterioration of the plant growth conditions due to climate change, and successive CAP reform and changes in the market^8^. The introduction of fodder crops may increase the fodder resources in those farms. Among them, *Lolium multiflorum* (Italian ryegrass) is an annual grass species widely used in dehesas, because of its favourable performance under their conditions, high herbage yield, excellent nutritive value and excellent re-growth allowing for multiple harvests during its growth cycle^6^.

Therefore, to maintain the ecological, social and cultural importance of dehesas and other agrosystems with extensive management and low incomes, identifying alternatives that increase productivity but allow for conservation by decreasing the use of synthetic chemical products is critical. One possible approach to achieve this goal comes from the application of effective biopesticides or biostimulants. The use of these bio-products may contribute to reaching the target of the Farm-to-Fork Strategy of the European Union of a 50% decrease in the overall use and risk of chemical pesticides by the year 2030. Great interest in such products arose from the creation of the European Biostimulants Industry Council in 2011, which encompasses (as of late 2022) more than 69 companies and organizations. These bio-products, which are included in EU Regulation 2019/1009, can be classified as microbial or non-microbial products^9^. Although several of these bio-products are already in commercial use, the vast potential of microorganisms to be exploited for this purpose remains to be explored. One of the first steps in obtaining a microbial bio-product is identifying novel microorganisms with the desired properties.

Endophytic fungi, microorganisms that colonize internal plant tissues without causing apparent disease symptoms^10^, might be suitable candidates, because they have been found to confer adaptive advantages or resistance toward both biotic^11^ and abiotic stresses on their hosts^12^. Different species of fungal endophytes have already been described as growth promoters in their hosts^13,14^. In this regard, such a mutualistic relationship may favour key changes in plant physiology, including phytohormonal content, or modifying nutrient absorption and soil conditions in the rhizosphere^15,16^. The main mechanisms, activities or principles involved in the growth promotion effects of fungal endophytes on their hosts have been summarized in several review articles^17,18^. Fungal endophytes promote the synthesis of siderophores^19,20^, increase nitrogen fixation^21^, increase phosphate solubilization^22,23^, suppress ethylene by 1-aminocyclopropane-1-carboxylate deaminase (ACC deaminase)^24^, and affect the synthesis of enzymes^25,26^. Fungal endophytes may also influence plant development through the production of phytohormones^22,27^ such as indole 3-acetic acid (IAA) and gibberellins (GA)^28^. IAA, the main auxin in plants, regulates several developmental traits of organogenesis^29^. GAs are terpenoids that were first identified in cultures of the fungus *Gibberella zeae* (currently known as *Fusarium fujikuroi*)^30^ and perform fundamental functions in plant development^28^. In addition, a wide variety of other secondary metabolites, such as exopolysaccharides or phenols, may promote plant growth through their antioxidant activity ^31,32^.

The potential of fungal endophytes isolated from plant species growing in dehesas to serve as plant growth promoters or protectors, which may increase herbage productivity and nutritive value, has been demonstrated in several fodder crops, such as *Trifolium subterraneum*^33^, *Ornithopus compressus*^34^ and *Lolium rigidum*^35^. Three endophytic isolates, E060, E025 and E051, have shown clear growth promoting activity toward the plants on which they were applied. In all cases, the increase in plant productivity produced by the endophyte was observed after artificial inoculation into their in a greenhouse under strictly controlled conditions. However, multiple attempts to reproduce these positive effects under field conditions have produced inconsistent results, because the establishment of the relationship and the induction of the desired effects may be strongly influenced by the environment and other external conditions, such as interactions with other microorganisms ^36^. To overcome this problem, bioactive compounds produced by these fungi might be applied directly, because those compounds have been found to be responsible for the beneficial effects in many cases^37,38^. These secondary metabolites, which can be obtained under controlled conditions, might not be as strongly affected by the environmental conditions after application, and their handling and application by farmers might be much easier than applying living organisms. Therefore, although the direct application of such bioactive secondary metabolites has been much less studied than inoculation with living organisms^14,35^, the suitability of the compounds produced by fungal endophytes isolated from dehesas plants to protect plants against several phytopathogens and promote plant growth, has already been demonstrated in *Lupinus luteus*^39^.

To correctly identify the strains to be used in this study, multiple genetic loci were examined to ensure the confidence of the identification. In addition, to identify novel compounds with plant growth promoting activity that would be suitable for further use and development of potential commercial bio-products, the following hypotheses were made: (1) the growth promoting activity, already observed in the fungal endophytes E025, E051 and E060 after their artificial inoculation in plants^33,34,40^, is caused by the secondary metabolites that they may produce, and (2) the application of those secondary metabolites, produced previously in vitro, promotes growth in plants. On the basis of these hypotheses, the objectives of the present study were (i) to determine the nature and/or the activity of the compounds produced by these three fungal endophytes, E025, E051 and E060, in the most common traits associated with growth promotion and (ii) to evaluate the effects of fungal compounds on plant-growth promotion features after their application to seeds and seedlings. For these purposes the forage crop *Lolium multiflorum* was used as a model plant. The taxonomic place of the three fungal endophytes studied was also performed, and the main metabolites contained in the fungal compounds were tentatively identified.

## Materials and methods

### Identification of fungal strains

The three fungal endophytic strains used in the present study, E060, E025 and E051, were isolated as explained in Santamaria et al.^41^ from healthy plants collected from dehesas in the Extremadura region in the southwest of Spain. Specifically, both E060 and E025 were isolated from leaves of *Biserrula pelecinus*, and E051 was isolated from leaves of *Ornithopus compressus*. The strains were selected on the basis of their ability to positively affect the growth of their hosts after artificial inoculation, as observed in previous studies and in preliminary unpublished assays^33,34,41^. In those preliminary assays, many other strains tested did not show any positive activity in plant growth promoting traits, or they were very limited (data not shown). The endophyte E060 was previously identified as *Fusarium* sp., according to the conidial morphology and its internal transcribed spacer (ITS) region by Santamaria et al.^40^. In the present study, two additional loci, the second largest subunit of RNA polymerase II (r*pb2*) and β-tubulin (*tub2*), were also used to specifically identify this species. The identification of the endophyte E025 was based on morphological and molecular procedures, by analysis of the 28S ribosomal RNA (LSU) and ITS regions. For the morphological characteristics of E025, fertile fungal structures grown in oatmeal agar medium were mounted and measured in lactic acid, with at least 30 measurements of each structure. Photomicrographs were obtained with a Nikon Eclipse Ni compound microscope, a DS-Fi3 digital camera (Nikon, Tokyo, Japan) and NIS-Elements imaging software v. 5.20. Finally, the endophyte E051 was identified through molecular procedures by analysis of the ITS, LSU and *tub2* regions. For the molecular procedures, in all cases, DNA was extracted and purified directly from colonies growing in yeast malt (YM) agar (malt extract 10 g L^−1^, yeast extract 4 g L^−1^, D-glucose 4 g L^−1^ and agar 20 g L^−1^, pH 6.3 before autoclaving) according to the Fungal gDNA Miniprep Kit EZ-10 Spin Column protocol (NBS Biologicals, Cambridgeshire, UK). The amplification of the loci analysed (ITS, LSU, *rpb2* and *tub2*) was performed according to White et al.^42^ and Vilgalys and Hester^43^. PCR products were purified and sequenced with Sanger sequencing at Microsynth Seqlab GmbH (Göttingen, Germany). Consensus sequences were obtained with Geneious® 7.1.9 (http://www.geneious.com,^44^). The obtained regions were compared with sequences from the GenBank (www.ncbi.nlm.nih.gov) database with a BLAST search.

Additionally, for endophyte E051, because the BLAST search was inconclusive, a phylogenetic analysis was performed on the basis of the combination of the three loci sequences (ITS, LSU and *tub2*) and selected members belonging to the family Xylariaceae, with *Biscogniauxia nummularia* MUCL 51,395 and *Graphostroma platystomum* CBS 270.87 (both members of the sister family Graphostromataceae) as outgroups. GenBank accession numbers of the sequences used in the phylogenetic study are shown in Table S1. Each locus was aligned separately with MAFFT v. 7^45^ and manually adjusted in MEGA v. 10.2.4^46^. Individual gene phylogenies were verified for conflicts before the three locus datasets were concatenated^47,48^. The maximum-likelihood (ML) and Bayesian inference analyses including the three loci were performed as described by Harms et al.^49^. The best evolutionary model for each sequence dataset was calculated with MrModeltest v. 2.3^50^. Bootstrap support (bs) ≥ 70 and posterior probability values (pp) ≥ 0.95 were considered significant^51^.

### Filtrates and crude extracts production from fungal endophytes

To work with the compounds produced by the endophytes, we obtained two sample types: filtrates and extracts. The filtrates consisted of the culture medium (yeast malt extract, YM) in which the fungus had grown under specific conditions, after removal of the fungal structures. When those filtrates were extracted with ethyl acetate and evaporated, the ‘dry crude extract’ (or ‘dry extract’) was obtained. When this crude extract was dissolved again in sterilized distilled water at a specific concentration depending on the experiment, the dissolved extract was obtained. Because the dry crude extract was used only for storage, we use the term extract herein to refer to dissolved crude extract. To obtain the filtrates and extracts for the different assays, we collected mycelium samples of each endophytic species from 7-day old colonies actively growing in Petri dishes with potato dextrose agar (Oxoid Microbiology products) supplemented with chloramphenicol. Each endophyte was incubated in 500 mL flasks containing 250 mL of YM broth at 23 °C and 140 rpm until 2 days after the glucose in the medium was consumed. The culture was then filtered through (ø = 0.2 μm) sterile paper discs to separate the mycelium from the liquid phase (filtrate). To obtain the crude extracts, as described above, we extracted this filtrate with methods described by Halecker et al.^52^, with an equal amount of ethyl acetate, then evaporated the solvent with a rotary rotavapor (Hei-Vap ML/G1). After complete evaporation, crude extract was dissolved in methanol and concentrated (SpeedVac concentrator, Eppendorff) until completely dry.

Filtrates were used for the quantification of the phytohormone-like substances, and the germination tests and plant growth promotion tests under growth-chamber conditions. Extracts were used for the determination of the antioxidant activity and for the plant growth promotion tests under greenhouse conditions.

### Determination of plant growth promoting traits of fungal compounds

The potential of the three endophytic fungi to produce substances with plant growth promotion activity was determined according to the following traits commonly associated with growth promotion: (i) production of phytohormone-like substances; (ii) antioxidant activity, together with the synthesis of phenolic compounds; and (iii) mobilization of nutrients through phosphate solubilization ability, compounds such as siderophores, or ammonia production.

#### Production of phytohormone-like substances

Phytohormone-like substances in the filtrates were determined. The auxin-like substances were estimated through determination of the IAA content in the fungal filtrates by colorimetric tests^53^. First, 1 mL of each filtrate was mixed with 2 mL of Salkowski reagent. Subsequently, samples were vortexed for 1 min and incubated in the dark for 30 m, after which the absorbance was measured at 530 nm (JP Selecta UV 3100). The concentration of IAA was calculated from the regression equation of a standard curve of pure indole-3-acetic acid (Sigma Aldrich). In addition to growth in standard conditions, endophytes were grown in 50 mL of YM medium supplemented with 5 mM L-tryptophan (Panreac), the main precursor of IAA. The production of gibberellin-like substances was also estimated as gibberellic acid equivalents (GAE) by colorimetry, as described by Holbrook & Edge^54^. Briefly, 15 mL of the filtrate was mixed with 2 mL of 21.9% zinc acetate, followed 2 min later by 2 mL of 10.6% potassium ferrocyanide; the mixture was then centrifuged at 2000 rpm for 15 min. A 5 mL volume of 30% HCl was added to the same amount of the supernatant before incubation at 20 °C for 75 min. Finally, absorbance was measured at 254 nm and gibberellic acid (GA_3_, Sigma Aldrich) was used as standard for quantification. All samples were analysed in triplicate, and the results are expressed as mg of compound (i.e., IAA or GAE, respectively) per mL of filtrate.

#### Determination of antioxidant activity

Antioxidant activity was determined in fungal extracts, as estimated through a DPPH (1,1- diphenyl-2-picrylhydrazyl) assay, according to the procedure described by Liu et al.^55^ with slight modifications. Samples were prepared as mixtures of 2.9 ml of 0.004% aqueous DPPH and 0.1 mL (3 mg mL^−1^) of each fungal extract. These samples were incubated in the dark at 25 °C for 30 min, and their absorbance was immediately measured at 517 nm with a spectrophotometer (JP Selecta UV 3100). The DPPH scavenging ability was calculated with the following equation: *% radical scavenging* = (absorbance control—absorbance sample)/(absorbance control)*100. All samples were analysed in triplicate.

#### Total polyphenols content

The total polyphenol content (TPC) of the extracts was estimated with the Folin-Ciocalteu method^56^, with gallic acid as a reference: 1 mL (1 mg mL^−1^) of extract was mixed with 500 μL of 50% aqueous Folin–Ciocalteau reagent, 1.5 mL of 20% aqueous Na_2_CO_3_ and 2 mL of distilled water. The samples were then incubated at room temperature in the dark for 30 m, and the absorbance was subsequently measured at 765 nm. All samples were analysed in triplicate. The polyphenol content is expressed in mg of gallic acid equivalents per g of fungal extract.

#### Determination of nutrient mobilization

Nutrient mobilization was evaluated by analysing three parameters: phosphate solubilization ability, siderophore production and ammonia production of each endophyte. For estimation of phosphate solubilization ability, an actively growing plug of each endophyte was sown in a Petri dish with National Botanical Research Institute phosphate (NBRIP) growth medium with 1.5% agar^57^. Plates were incubated for 7 days at 27 °C, and the presence of a clear halo around the colony was verified. This area was measured to estimate the solubilization ability of the endophyte through the following formula: *solubilization index (%)* = *(colony diameter* + *clear zone diameter)/(colony diameter)*^58^. Three replicates of each endophyte were assessed.

Siderophore production was estimated with the method described by Pérez-Miranda et al.^59^, which is a modification of the Chrome Azurol S (CAS) universal assay. An actively growing plug of each endophyte was sown in the centre of a Petri dish with 20 mL of Minimal Medium 9 and incubated in a growth chamber at 27 °C for 7 days. After 7 days, 1 L of a modified CAS solution without nutrients was prepared with 60.5 mg CAS, 72.9 mg HDTMA, 30.24 g PIPES, 1 mM FeCl_3_·6H_2_O in 10 mL of 10 mM HCl and agarose (0.9%, w/v). After being cooled to 50 °C after autoclaving, 10 mL of the solution was poured on each Petri dish with Minimal Medium 9 containing the 7 day-old fungal colonies. After 15 min, a colour change was observed in the CAS medium surrounding siderophore producing microorganisms. This halo was measured with the same formula as in the case of phosphate solubilization. Additionally, the protocol was repeated with a non-deferrated medium to demonstrate that no siderophore production was induced under normal conditions. For both assays, a blank was introduced with a plug of uninoculated potato dextrose agar medium. Three replicates of each endophyte were assessed.

Finally, the ability of the endophytes to produce ammonia was qualitatively assessed by growing each species in peptone water for 72 h at 28 °C and subsequent addition of 1 mL Nessler’s reagent. A colour change to faint yellow indicated minimal ammonia production, whereas a deep yellow to brownish colour indicated maximal ammonia production^58^. Three replicates of each endophyte were assessed.

### Effects of filtrates on seed germination and seedling growth of *Lolium multiflorum*

The potential of the fungal endophytes as plant growth promoting organisms (i.e. bio-stimulants) was first evaluated by studying the influence of their filtrates on seed germination and seedling growth in *Lolium multiflorum*, a model plant important in forage production. The *L. multiflorum* plants used in the experiment were obtained from seeds purchased from commercial sources (Cv Locobello from Batlle SA, Barcelona, Spain). To evaluate this plant growth promotion potential effect, we performed two experiments. In the first experiment the filtrates were applied on the seeds; in the second one, the filtrates were applied on the seedlings. For both assays, seeds were first surface disinfected by immersion in a solution of 2% sodium hypochlorite with two drops of Tween-80 for 2 min and washing them three times with sterile distilled water. For the seed experiment, 60 surface disinfected seeds were soaked in 2 mL of either filtrate or control treatments for 6 h (12 seeds per treatment). Two control treatments were used—sterile distilled water and YM medium—to evaluate any eventual effects of YM medium on the studied parameters. Subsequently, seeds subjected to each treatment were randomly divided in three groups (replicates) of four seeds each and placed in Petri dishes between two sterile filter paper discs with 5 mL of sterile distilled water. Plates were then kept in a growth chamber with 16 h of light at 30 °C and 8 h of darkness at 20 °C. The germination percentage was controlled during the following 15 days. After that period, five seedlings per treatment were randomly selected and the following growth parameters were determined: main shoot and longest root length, number of main roots and plant dry matter measured after drying the seedlings at 60 °C until a constant weight was reached. The vigour index induced by the filtrates was also determined according to Islam et al.^60^ as follows: *vigour index* = % of final germination * plant shoot elongation (cm).

For the seedling experiment, surface disinfected seeds of the same *L. multiflorum* cultivar were first sown into 7 × 7 × 6 cm plastic pots (one seed per pot) containing an autoclaved mixture of 1:1 (v/v) commercial substrate (pH 7.0 ± 0.5; EC 1.5 ± 0.1 dS m^−1^; organic matter 60.0 ± 2.0%; apparent density 0.5 ± 0.1 kg L^−1^; N 1.29 ± 0.08%; P_2_O_5_ 0.58 ± 0.05%; K_2_O 1.25 ± 0.10%; and MgO 0.46 ± 0.04%; COMPO SANA Universal, COMPO GmbH & Co. KG, Münster, Germany) and perlite, and then kept in a growth chamber with 16 h of light at 30 °C and 8 h of darkness at 20 °C. When the seedlings were 15 day-old, filtrates were applied according to a procedure described by Ismail et al.^61^, with slight modifications: 600 µl of fungal filtrates were applied to the tip of the shoot with five pots (replications) per treatment, then kept in the growth chamber under the same conditions. In addition, sterilized distilled water and YM medium instead of fungal filtrates were used as controls. After 15 days of application, the plants were harvested, and the following parameters determined: main shoot and longest root length; number of main roots; plant dry matter, measured after drying the seedlings at 60 °C until a constant weight was reached; and photosynthetic activity, estimated with a SPAD meter (SPAD 502 Plus Chlorophyll Meter, Konica Minolta Inc., Tokyo, Japan).

### Effect of the extracts on plant growth of *Lolium multiflorum* under greenhouse conditions

To evaluate the plant growth promoting activity of the fungal compounds present in the extracts in a more uncontrolled environment, they were applied to *Lolium multiflorum* plants under greenhouse conditions. The relative humidity and temperature recorded in the greenhouse during the experiment are presented in Fig. S1. In late September of 2019, three surface disinfected seeds per pot were directly sown in 7 × 7 × 6 cm plastic pots, containing the same mixture of 1:1 (v:v) substrate and perlite. The plants were grown in a greenhouse with the necessary irrigation to keep them at field capacity. During the test, plants did not receive any fertilizer and were maintained with sufficient separation to avoid contact between them, to avoid cross-results. Before the foliar application treatments, plants were thinned to one plant per pot, to evaluate the effects of the extracts on the tillering rate. Treatments were applied when the plants were 1 month old by spraying with 1 mL of 3 mg mL^−1^ dry extract solution in water per pot. A unique control treatment with water, the solvent used to dissolve the extracts, was used. For each of the four treatments studied (i.e., each of the three fungal extracts and a sterile distilled water-control), 15 replications were performed. The treated plants were kept in the greenhouse for 1 month. At this moment, just before harvesting, photosynthetic activity was estimated through chlorophyll measures with a SPAD meter. Immediately after harvesting, number of plants per pot, the number of main roots per plant, and main shoot and longest root length were measured. The dry weights of the shoot and root parts of each plant were also measured after they had been dried in an oven at 60 °C until a constant weight was reached.

### HPLC analysis of the fungal extracts

To identify the metabolites in the fungal extracts, a mass spectrometry was performed on an Agilent 6520 Accurate Mass Q-TOf LC/MS system (Agilent, Santa Clara, CA, USA) equipped with an electrospray ionization interface, and operated in positive ion mode with parameters set as follows: capillary voltage, 3500 V; fragmenter, 100 V; pressure of nebulizer, 35 psig; drying gas temperature, 300 °C; acquisition range 150–800 m/z. Nitrogen was used as the drying gas at a flow rate of 12.0 L min^−1^. The system was also equipped with a diode array detector operating from 280 to 350 nm with a step of 2 nm. Samples were eluted on an Agilent Zorbax eEclipse Plus C18 Rapid Resolution column (4.6 × 100 mm 3.5 μm). The column temperature was maintained at 30 °C. A mobile phase consisting of 0.1% formic acid in ultrapure water (obtained from Millipore Integral-5 purification system) (solvent A) and 0.1% formic acid in acetonitrile (solvent B) was applied with the following gradient: 0–10% B (0 min), 10–100% B (30 min), 100% B isocratic mode (10 min) and, for column re-conditioning 100–10% B (1 min) and 10% B (7 min). Both formic acid and acetonitrile were of LC/MS grade. The flow rate was set at 0.30 mL min^–1^ and the injection volume was 1 μL.

### Statistical analysis

The effects of the fungal treatment on each test were evaluated withy one-way analysis of variance (ANOVA) when the normality (Shapiro–Wilk test) and homogeneity of variance (Levene’s Test) criteria were verified. Fisher’s protected least significant difference (LSD) test for multiple comparison was used when significant differences (*P* < 0.05) were found in the ANOVA. All analyses were performed with the Statistix v. 8.10 package (Analytical Software, USA). All methods were performed in accordance with relevant local/national/international guidelines and regulations.

## Results

### Molecular phylogeny and taxonomy

For the endophyte E60, the BLAST search of NCBI´s GenBank nucleotide database with *rpb2* and *tub2* sequences indicated that this strain was *Fusarium avenaceum* (*rpb2* showed 100% nucleotide similarity with the neotype strain *F. avenaceum* NRRL 26911 [MG282401], and *tub2* showed 100% similarity with different strains of *F. avenaceum* [e.g., EU357845 and EU357851]). The endophyte E025 was identified as *Sarocladium terricola*, according to a BLAST search with ITS and LSU sequences. Both sequences showed 100% similarity with *S. terricola* CBS 243.59 (ITS and LSU GenBank accession numbers HG965040 and MH869389, respectively).

**Figure 1:**
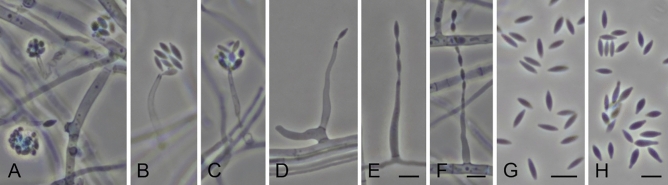
*Sarocladium terricola* (E025). (**A–C**). Conidiophore and conidia arranged in slimy masses. (**D–F**). Conidiophore and conidia in chains. (**G**), (**H**). Conidia. Scale bars: 5 μm; the scale bar of (**E**) applies to images from (**A** to **E**).

The molecular identification of E025 was supported by the morphological analyses, which showed reproductive structures similar to those described by Giraldo et al. ^62^ for *S. terricola*, including phialidic conidiophores with distinct periclinal thickening at the conidiogeneous locus, and fusiform, smooth-walled, hyaline conidia (Fig. 1). However, our strain produced conidiophores with a wider range of sizes [8–30(–47) μm long and 1.5–3 μm wide at the base vs. 12–30(–35) μm long and 1–2 μm wide at the base] and shorter conidia [3–5.5 μm vs. 4–7(–8) μm].

**Figure 2:**
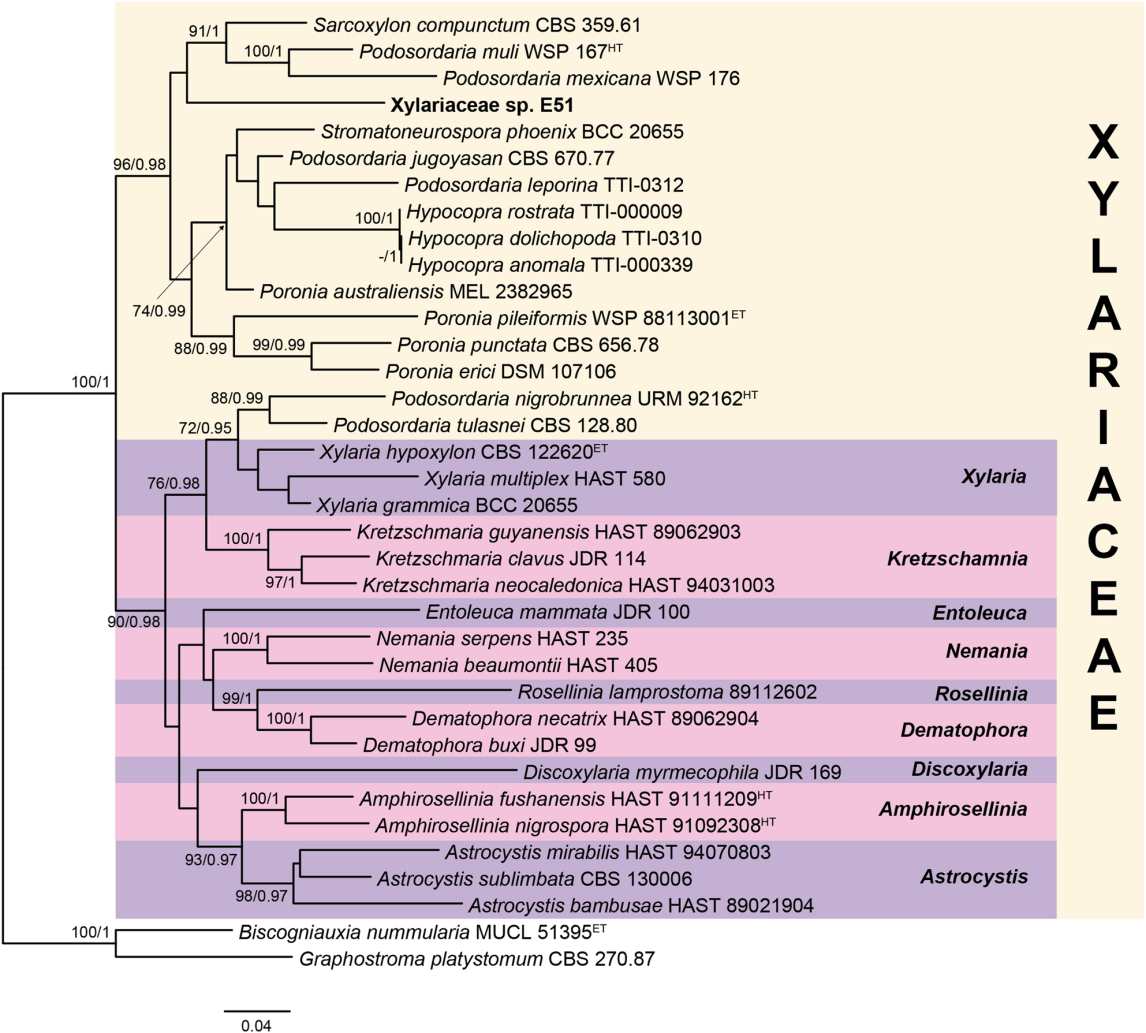
RAxML phylogram obtained from the combined ITS, LSU and *tub2* sequences of selected strains belonging to the family Xylariaceae. *Biscogniauxia nummularia* MUCL 51395 and *Graphostroma platystomum* CBS 270.87 were used as outgroup. Bootstrap support values ≥ 70/Bayesian posterior probability scores ≥ 0.95 are indicated along branches. Branch lengths are proportional to distance. Strain studied in the present work is indicated in **bold**. Ex-epitype and holotype strains of the different species are indicated with ^ET^ and ^HT^, respectively.

The ITS, LSU and *tub2* sequences of the endophyte E051 showed low nucleotide similarity with taxa belonging to the family Xylariaceae. To achieve a proper identification at the genus and species level, a phylogenetic study was performed. The lengths of the individual alignments used in the combined dataset were 536 bp (ITS), 816 bp (LSU) and 1436 bp (*tub2*), and the final total alignment was 2788 bp. The most likely tree obtained from the RAxML analysis of the combined dataset including RAxML bootstrap support and Bayesian posterior probability at the nodes is shown in Fig. 2. The isolate E051 was located in the well-supported clade (100% bs/1 pp) representing the family Xylariaceae. This isolate formed a well-supported subclade (96% bs/0.98 pp) together with several species of *Hypocopra*, *Poronia, Podosordaria*, *Sarcoxylon* and *Stromatoneurospora*. The lack of type material of the available type species of these genera, does not allow us to verify whether our strain may belong either to any of them or to a new one. The lack of sporulation of our isolate prevented also any morphological approach. Therefore, we decided not to assign the isolate E051 to any taxonomic category below the family level until new information can be obtained. The final taxonomic assignment and basic information for the endophytes used in the present study according to the available data are shown in Table 1.

**Table 1:** Taxonomic assignment of the endophytic fungi used in the experiments.

Code	Plant host	Taxonomic assignment	GenBank accession #			
			ITS	LSU	*tub2*	*rpb2*
E025	*Biserrula pelecinus*	*Sarocladium terricola*	OK161081	OP019605	–	–
E051	*Ornithopus compressus*	Xylariaceae sp*.*	OK161080	OP019604	OP856689	–
E060	*Biserrula pelecinus*	*Fusarium avenaceum*	KP698325	–	OP856691	OP856690

### Plant growth promoting traits of fungal compounds

The results for the evaluated traits, commonly associated with plant growth promotion (production of phytohormone-like substances; antioxidant activity and synthesis of phenolic compounds; mobilization of nutrients through phosphate solubilization ability; and production of siderophores and ammonia) are summarized in Table 2.

**Table 2:** Plant growth promoting traits of the fungal endophytes. A summary of the one-way ANOVAs is also shown, indicating the degrees of freedom (DF), *F* values and *P*-values.

Trait		*F*	*P*-value	*DF*	*Sarocladium* *terricola*	Xylariaceae sp.	*Fusarium* *avenaceum*
Phytohormone	IAA (µg mL^−1^)	15.68	0.004	2	6.33 ± 0.20 a	5.70 ± 0.02 b	6.61 ± 0.02 a
	IAA + (µg mL^−1^)	493.95	< 0.001	2	19.44 ± 0.22 a	13.69 ± 0.01 b	13.56 ± 0.14 b
	GA_3_ (µg mL^−1^)	67.73	< 0.001	2	396.67 ± 0.09 b	449.66 ± 4.19 a	446.30 ± 4.63 a
Antiox. Activity	TPC (mg GAE g^−1^)	867.43	< 0.001	2	77.59 ± 0.06 b	108.06 ± 1.75 a	29.82 ± 1.53 c
	DPPH (%)	1186.48	< 0.001	2	54.66 ± 0.25 b	54.11 ± 0.25 b	66.47 ± 0.18 a
Nutrient Mobilization	Siderophore (cm)	37.98	< 0.001	2	1.77 ± 0.25 a	0.00 ± 0.00 c	1.19 ± 0.01
	Phosphate (cm)	137,641	< 0.001	2	0.00 ± 0.00 b	1.24 ± 0.00 a	0.00 ± 0.00 b
	NH_3_				+	–	+ + +

IAA: indole acetic acid; IAA + : indole acetic acid supplemented with L-tryptophan; GA_3_: gibberellic acid; TPC: total polyphenol content; DPPH: % of scavenging of DPPH; siderophore: halo of siderophore production; phosphate: halo of phosphate solubilization; NH^3^: production of ammonia (− no production; + low production; +  +  + high production). For each quantitative parameter, averages in the same row with different letters are significantly different according to the LSD test. Data are expressed as mean ± standard error (n = 3).

Regarding the phytohormone-like substances, the three endophytes showed an ability to produce both indole acetic acid and gibberellin-like substances, according to analysis of their filtrates. For IAA, the response was positive in both culture media, although the concentration was markedly higher (2–threefold) when the medium was supplemented with L-tryptophan. The IAA concentration was significantly higher in the filtrate of *S. terricola* in the test with enriched medium, whereas the contents for this endophyte and* F. avenaceum* did not differ in the test with standard medium. In contrast, the filtrate of Xylariaceae sp*.* contained the highest concentration of gibberellic acid, although no significant differences with respect to *F. avenaceum* were observed (Table 2).

Regarding antioxidant activity, the extract of the endophyte Xylariaceae sp*.* produced the highest amounts of phenolic compounds. However, this outcome did not result in higher DPPH scavenging ability. The extract of the endophyte *F. avenaceum*, which showed the lowest concentration of polyphenols, had the highest DPPH scavenging ability among the three fungi analysed (Table 2).

The nutrient mobilization results also varied significantly among fungi. Xylariaceae sp. was the only endophyte capable of solubilizing phosphate in the enriched NBRIP medium, but it produced neither siderophores nor ammonia. In contrast, the other two isolates showed opposite behaviour. Although *S. terricola* and *F. avenaceum* were unable to solubilize phosphates, both showed potential to produce siderophores and ammonia. Specifically, the endophyte *S. terricola* had a greater ability to produce iron chelators, whereas *F. avenaceum* generated a much more intense colour in the ammonia test, thus potentially indicating a greater ability to produce nitrogen compounds (Table 2).

### Effects of filtrates on seed germination and seedling growth of *Lolium multiflorum*

**Figure 3:**
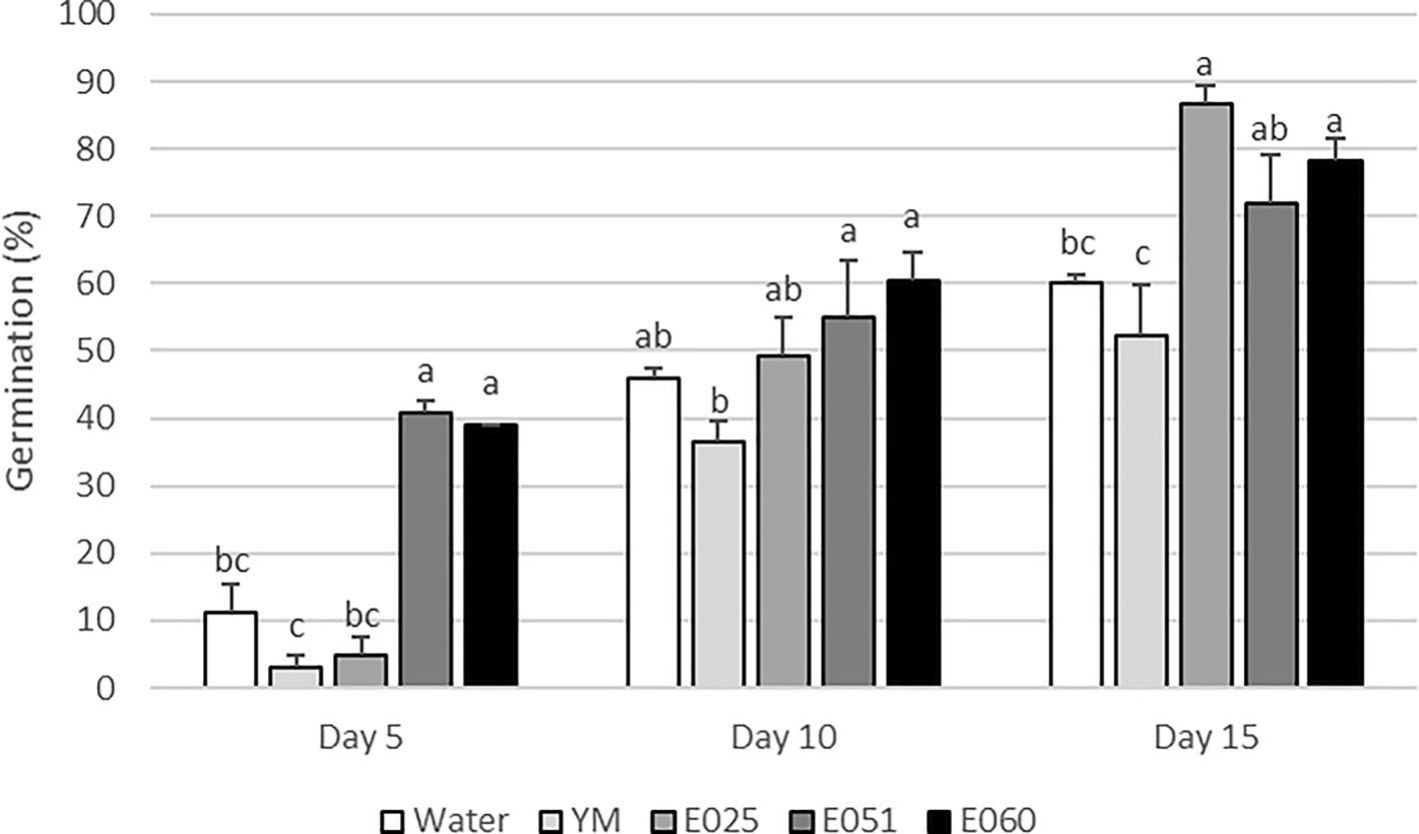
Effects of the fungal filtrates of each endophyte on the germination of *Lolium multiflorum* after 5, 10 and 15 days. Bars indicate means (n = 3) and error bars indicate standard error. For each evaluation day, different letters represent significant differences between means according to the LSD test (*P* ≤ 0.05). E025: *Sarocladium terricola*; E051: Xylariaceae sp; E060: *Fusarium avenaceum*; water: control with sterile distilled water; YM: control with yeast extract and malt culture medium.

In general, the three fungal filtrates had significantly positive effects in the development of *Lolium multiflorum* in both the seed and seedling assays. In the germination experiment no statistical difference was observed between the water and YM-medium controls, although some trend to be greater in water was observed (Fig. 3). The application of the three endophytic filtrates, compared with both controls, resulted in a significantly greater germination rate on the three evaluation days according to the ANOVA: day 5 (degrees of freedom, *df* = 4, *F* = 58.03, *P* < 0.001), day 10 (*df* = 4, *F* = 2.73, *P* < 0.05) and day 15 (*df* = 4, *F* = 7.55, *P* < 0.01). Compared with the control with YM-medium, which might be the most accurate in our case, the filtrates of the three endophytes, *F. avenaceum*, *S. terricola* and Xylariaceae sp., resulted in a greater germination rate of *L. multiflorum*, by 69%, 52% and 38%, respectively, at the end of the experiment after 15 days of evaluation. Except for *S. terricola*, the other two endophytes also resulted in a greater germination rate after 5 and 10 days of evaluation than did the YM-medium control (Fig. 3).

**Figure 4:**
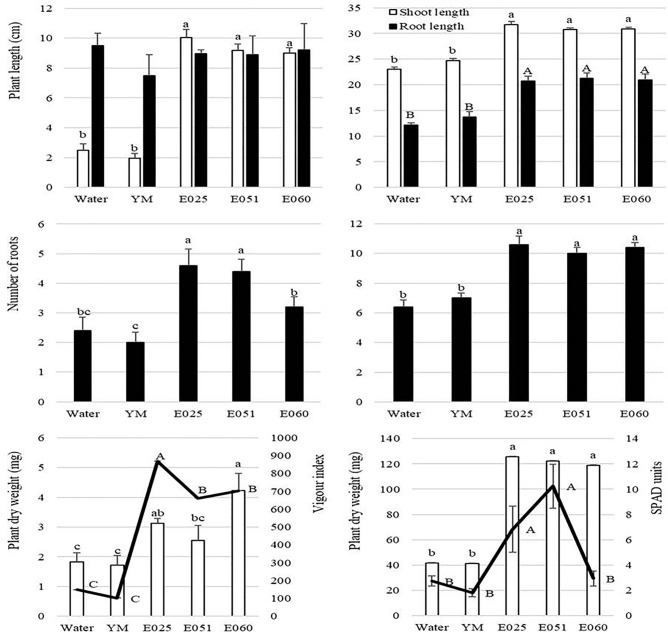
Effect of fungal filtrates on plant growth parameters in the *Lolium multiflorum* seed (**a**) and seedlings (**b**) assays. Data are expressed as mean ± standard error (n = 5). For shoot and root length, number of roots and plant dry weight, vertical bars indicate means and vertical lines, standard errors; for the vigour index and the Chlorophyll (SPAD units), the continuous lines indicate means and vertical lines, standard error. For each parameter, averages with the same letters (lowercase and uppercase were used to differentiate shoot and root length analysis, respectively) are not significantly different according to LSD test at a significance level of 0.05. YM: yeast malt extract broth culture-medium.

In this experiment, the application of the filtrates to the seeds also significantly increased (*P* ≤ 0.05) most of the growth traits in the seedlings (Table S2). Seedlings from seeds treated with any of the three filtrates showed significantly longer shoot parts (between 2.6 and 3.1-fold for *F. avenaceum* and *S. terricola*, respectively) than those treated with either water or YM-medium controls. The filtrates of *S. terricola* and Xylariaceae sp. also produced more roots per seedling (2.3 and 2.2-fold) and greater total dry weight (1.8 and 1.5-fold) than the controls (Fig. 4). The vigour index, which links the germination rate and the shoot development, was significantly higher for the three fungal treatments than the controls. The highest vigour index was found when the filtrate of *S. terricola* was applied; this was followed by the filtrates of Xylariaceae sp. and *F. avenaceum*, between which no differences were observed.

In seedling experiments in a growth chamber, the observed trends were very similar to those in the seed tests. Significant differences were found for the five measured parameters after applying the filtrates, compared with both water and YM controls. No significant differences were observed among the filtrates of the three endophytes for any parameter, except in the case of the SPADmeter, in which the best values were obtained with the filtrates of Xylariaceae sp. and *S. terricola* (Fig. 4).

### Effects of the extracts on plant growth of *Lolium multiflorum* under greenhouse conditions

For the greenhouse experiment, the seedlings were treated with the extracts, to complete and complement the information obtained with filtrates in the growth chamber essays. The results under greenhouse conditions with the extracts were consistent with those obtained in the growth chamber tests. Treatment of the seedlings with the extracts had significant positive effects (*P* < 0.05) on all variables studied, except for the SPAD measures (Table S3).

**Figure 5:**
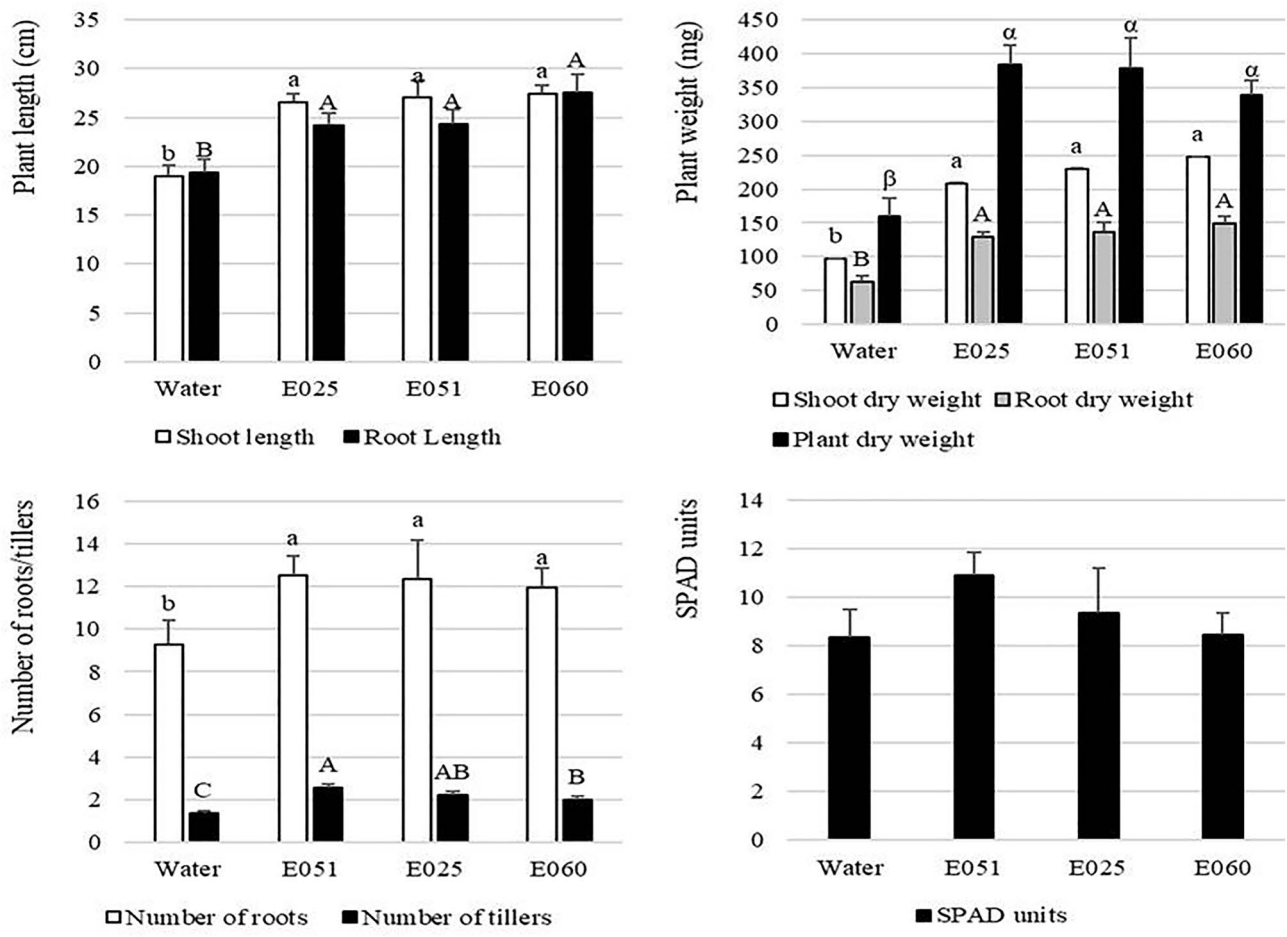
Effect of fungal extracts on plant growth parameters in *Lolium multiflorum* plants under greenhouse conditions. Data are expressed as mean ± standard error (n = 5). Vertical bars indicate means and vertical lines, standard errors. For each parameter, averages with the same letter (lowercase, uppercase, and Greek letters were used to differentiate shoot, root, and plant dry weight analysis, respectively) are not significantly different according to LSD test at a significance level of 0.05.

In all cases, compared to the controls, the three fungal extracts had positive effects on the development of the treated plants. Shoot length was between 40.05 and 44.54% greater when extracts of *S. terricola* and *F. avenaceum*, respectively, were applied. A similar trend was found for the root length, which showed at least a 25% greater length in plants treated with either Xylariaceae sp. or *F. avenaceum*, and a 42.27% greater length in plants treated with *S. terricola*, than the controls. Regarding dry weight, the seedlings treated with any of the extracts had shoot and root lengths at least twice those of seedlings treated with the controls (Fig. 5). The number of roots per plant was also higher (at least 28.5%) in seedlings treated with the extracts than the controls, and no significant differences were observed between endophytes. At harvest, the number of tillers per pot significantly differed among treatments, and the highest values were observed for pots treated with *S. terricola* extract, although no significant differences were observed with respect to treatment with the Xylariaceae sp. In both cases, the numbers of tillers per pot were much higher than those observed with control treatment.

### Tentative identification of metabolites by HPLC

As a first approximation to make a tentative identification of the metabolites responsible for the plant growth promotion effects observed, we analysed compounds present through HPLC of the fungal extracts. The chromatograms of the fungal extracts were compared with a library of phytohormones and phenolic compounds likely to be associated with plant growth promotion activity. The results of this preliminary analysis (Table 3) led to the identification of three compounds: two for the endophyte Xylariaceae sp. and one for *F. avenaceum*. No compound was identified in the crude extract of *S. terricola* with sufficient confidence to be included in this study. Further studies are needed to fully identify the compounds produced by these endophytes. The compounds tentatively identified from Xylariaceae sp*.* were the phytohormone zeatin and the polyphenol acetyl eugenol. In the case of *F. avenaceum*, the compound identified was gibberellin A2.

**Table 3:** Peak assignment for methanolic extracts of the endophytic fungi belonging to Xylariaceae sp. and *Fusarium avenaceum*.

Peak	Rt (min)	Obs. *m/z*	Target mass [charge 1]	Proposed formula	λmax (nm)	Proposed compound
Cpd. 1 (Xylariaceae sp.)	10.784	219.1115	219.112	C_10_H_13_N_5_O	350	Zeatin
Cpd. 20 (Xylariaceae sp.)	20.646	206.0954	206.0943	C_12_H_14_O_3_	350	Acetyl eugenol
Cpd. 2 (*Fusarium avenaceum*)	23.742	350.1735	350.1729	C_19_H_26_O_6_	280	Gibberellin A2

Rt: retention time; Obs m/z: observed mass/charge relationship.

## Discussion

In the future, farming systems will face problems such as climate change or restrictive agriculture policies, which might require increased efforts to improve both crop productivity and sustainability^63^. To achieve this goal, the use of beneficial microorganisms for sustainable crop production is becoming an option of interest^20^. The present study was aimed at contributing to this goal by revealing the potential of three fungi isolated from Spanish dehesas to promote plant growth, particularly via bioactive secondary metabolites. The utilization of microorganisms isolated from plants growing in the local agroecological conditions where they might be applied may be of interest, because it can favour their competitiveness against other native strains^22^. Furthermore, if endophytes are isolated from plants growing in harsh edaphoclimatic conditions, such as those in dehesas, they might be likely to confer plant growth promoting activity, because stressful conditions might favour the establishment of mutualistic plant-endophyte relationships. In fact, many fungal endophytes isolated from plants in dehesas have already been found to have activities of interest in improving plant production or protection^39,40^.

The present study identified (at different taxonomic levels) three fungal endophytes with plant-growth promoting activity: *Fusarium avenaceum, Sarocladium terricola* and Xylariaceae sp. *Sarocladium* (Hypocreales, Sordariomycetes) currently includes 28 species distributed worldwide^64–66^. Several *Sarocladium* species have been reported to be endophytes, mainly of members of Poaceae^64^, including *S. terricola*^67^. In the present study, *S. terricola* was isolated as endophyte of *Biserrula pelecinus* (Fabaceae) in Spain. To our knowledge, this is the first report of a species of *Sarocladium* isolated as an endophyte from a member of Fabaceae in Spain. The strain E051 was identified as a member of the family Xylariaceae, according to a phylogenetic study including ITS, LSU and *tub2* sequences. Unexpectedly, it was located in a lineage containing mostly coprophilous taxa belonging to the genera *Hypocopra, Poronia, Podosordaria, Sarcoxylon* and *Stromatoneurospora*. Within the extensive scientific literature on Xylariaceae and related families in the past decades, recent studies using polyphyletic approaches have revealed that many of these families have a saprotrophic and an endophytic phase in their life cycle^68^. Becker et al.^69^ have observed that coprophilous genera in Xylariaceae form an independent evolutionary lineage from their wood-inhabiting or endophytic relatives. The only non-coprophilous fungus located in this lineage is *Sarcoxylon*, a wood-inhabiting genus. However, little is known about its ecology, and very few specimens have been collected in the tropics^58^. The endophytic character of the isolate studied herein was clearly established, because it was isolated from apparently healthy plant material after a thorough surface disinfection^40^. Therefore, the strain E051 could also be considered a non-coprophilus species because it was isolated as an endophyte of *Ornithopus compressus*. Unfortunately, sporulation was not observed in the present study, which would have provided valuable information for more precise characterization of this isolate. Moreover, none of these genera are sufficiently well-delimited according to molecular data, because of the lack of type material of the respective type species. Therefore, further studies including more taxa of this lineage are needed to resolve the boundaries of these genera and to properly identify our isolate.

The strain of *F. avenaceum* used in the present study had already been shown to produce positive effects when it was artificially inoculated throughout a mix of spores and mycelia, although inconsistent results were observed among plant species. When it was inoculated on other members of the family Poaceae, such as *Poa pratensis*, it increased the uptake and accumulation of Fe, Ni and Zn in the herbage^41^. However, when it was applied as a living organism to Fabaceae, either *Trifolium subterraneum*^33^ or *Ornithopus compressus*^34^, the results were markedly different between those two legumes. The inoculation in *T. subterraneum* decreased the uptake of Al, Fe and Cr, and increased the fiber content of the forage, whereas in *O. compressus*, the concentrations of Pb, B, P, S and Zn, whereas that of Al, Fe and Pb, decreased. Regarding other growth traits, inoculation with this endophyte also produced an increase in the crude protein of the herbage, but only in the *O. compressus*, host species from which it had originally been isolated from^40^. Many of these aspects could be considered as contributing factors that may partially explain the plant growth promotion observed, although they were not studied in the present study. *Fusarium* includes plant pathogenic species^70^ as well as endophytes. This aspect is important because eventual pathogenic behaviour of endophytes in hosts after application might prevent further utilization for plant growth promotion. The *Fusarium* strain and the other isolates used in the present study have to date shown no pathogenicity when inoculated directly, or when its filtrates or extracts were applied.

The three endophytic isolates used in the present study indicated a clear and positive effect on the germination and seedling development of *L. multiflorum*, regardless of whether they were applied as filtrate or extract, to seeds or seedlings. The ability of endophytic fungi to positively influence the germination of different plant species has already been studied previously, although in most studies, endophytes were applied as living organisms, such as *Trichoderma* spp. in the germination of *Solanum lycopersicum*^71^, *Phaseolus vulgaris*^72^ or *Oryza sativa*^73^. Fewer studies have been performed in which the filtrate (or extract) was applied instead of the living organism to evaluate effects on germination, and the results have been diverse. For instance, a novel endophytic *Trichoderma* sp. has been found to produce metabolites that increase the germination of peanut seeds^74^. However, filtrates of *Epicoccum nigrum have* shown a neutral effect on the germination of *Lepidium sativum*^75^, although application of the culture medium as a control had a slightly negative effect on germination. That result was attributed to excess nutrients, which might have affected the seed’s osmotic balance. This possibility may explain why, in our results, the germination of *L. multiflorum* seeds when YM medium was used as control tended to present lower values than when distilled water was used. Also this fact may suggest that the effects caused by our endophytes on seed germination could be expected to be even more positive if metabolites had been applied without using a nutrient-rich medium, such as YM.

The presence of phytohormone-like substances, such as IAA and GA, in the filtrates of the endophytes studied might partially explain the improvements in germination rate and seedling growth. This possibility is supported by findings from other studies in which the external addition of gibberellins induced the production of hydrolytic enzymes such as amylase^76^, which might hydrolyze starch into simple sugars, thus enhancing seedling development^77^. The production of GA by our endophytes was confirmed by the HPLC analyses, in which GA_2_ was detected in the extract of *F. avenaceum*. IAA has also been considered a catalytic factor in the colonization process of hosts by endophytes^78^ and my indirectly favour their potential plant growth promoting activity. In general, the IAA produced by the endophytes in the present study was similar to that in other studies, such as Waqas et al.^79^, in which the production ranged between 3.89 and 29.8 μg mL^−1^, or Bader et al.^22^, in which the strains with the highest ability to produce IAA had values between 13.2 and 24.3 μg mL^−1^. The obtained IAA values under standard conditions, which could already be considered as quite acceptable, became at least doubled when tryptophan was added. This finding was expected, on the basis of prior literature^80^: tryptophan is the main precursor of this phytohormone^81^. In the HPLC analyses, the phytohormone zeatin was also detected in the extract of Xylariaceae sp. This compound had already been found to affect the growth of rice plants under hyperthermia conditions^82^. In the greenhouse experiment in our study, the maximum temperature in late September to early October was quite high (Fig. S1). Therefore, the application of extracts including zeatin could have benefited treated plants. Nevertheless, since zeatin was not found in the extracts of the other two strains, which also provided growth promotion, further studies should be specifically designed to elucidate the actual role of this compound in such an effect.

The improvements in the shoot and root length, dry weight, and number of roots and tillers might also be explained by the phytohormone-like substances produced by endophytes, and by the presence of other secondary metabolites influencing growth, for example, for *S. terricola*, for which the compound cyclo(L-Leu-L-Trp) was tentatively detected in its crude extract, or *F. avenaceum*, which appeared to produce peniperquinolone (data not shown). Both compounds have been found to promote plant growth when produced by other fungal species, such as *Penicillium*^83^. Additionally, the presence of organic acids in the filtrates, which could have decreased the pH in the substrate, might have favoured the assimilation of nutrients such as phosphorus^79^ or the presence of small amounts of ammonia that might promote plant development^80^. Both explanations might be supported by our results: Xylariaceae sp*.* solubilized phosphates, whereas *F. avenaceum* and *S. terricola* produced ammonia. These traits may partially explain the higher root growth in seedlings treated with the filtrates of these three endophytes.

Other compounds, such as polyphenols, which have antioxidant activity, could also influence plant development^84^. In the present study, the antioxidant activity of the extracts was determined through their DPPH radical scavenging potential, as well as the TPC. The amounts of DPPH produced by the endophytes studied herein were similar to those reported for other endophytes considered to have antioxidant activity^56^, although DPPH scavenging potential values often vary between 20 and 70% depending on the endophytic strain^85^. However, the relationship between antioxidant activity and polyphenol concentration might not be very clear, giving that in several cases, such as in the extract of *F. avenaceum*, a high value for the scavenging of DPPH radicals did not corresponded to lowest high value of TPC. This fact might have been due to the presence of other non-phenolic compounds with antioxidant ability in the extract, such as carotenoids, which were not evaluated in the present study, or polysaccharides that have been demonstrated to have antioxidant activity when generated by different *Fusarium* species, such as *F. oxysporum*^86^. In the present study, total polyphenols were highly produced by the endophyte Xylariaceae sp., in agreement with findings from other studies in which other species of this family have been found to have high concentrations of TPC^87^. Supporting this aspect, acetyl eugenol, compound which has been found to be an antioxidant and to have biocontrol activity^88^, was also detected in the extract of Xylariaceae sp. by HPLC.

However, polyphenols might have been found affecting plant growth mainly by means of environmental stress defense and protection against multiple pests and diseases^89^. Therefore, since in the present study, experimental conditions were quite favourable for plant development, because plants were watered regularly, and no clear pest and diseases attacks were observed, this protective role of polyphenols might not have been the predominant mechanism in our case. Nevertheless, other studies have suggested an important role of polyphenols in plant growth promotion through several other mechanisms. For instance, polyphenols have been found to aid in cell wall formation, growth regulation, and leaf movement or germination^84^. This other mechanisms could also partially explain why the exogenous application of filtrates or extracts containing phenols in the present study might have positively affected the growth parameters of the *L. multiflorum* plants. Nevertheless, further studies on these compounds should be performed to corroborate these expectations.

Finally, the improvements in the growth parameters of the *L. multiflorum* plants might also be explained by the nutrient mobilization activity observed, particularly in two of the endophytes, *F. avenaceum* and *S. terricola*. Both endophytes showed a high potential to produce both siderophores and ammonia in the in vitro tests. The ability to produce ammonia might explain the effects of both fungi in increasing both shoot and root elongation, by enhancing the accumulation of nitrogen in the plant through nitrification^90^. If this possibility is further confirmed in specifically designed research, their application might decrease fertilization needs, in accordance with the objectives of the new European strategy for agriculture ‘Farm-to-Fork’. The production of siderophores, in contrast, might increase iron absorption, but also could act as a biocontrol agent, by decreasing the accessibility of iron to pathogens^91^. The potential to solubilize phosphates in vitro was observed for only the endophyte Xylariaceae sp. This activity is often associated with the production of organic acids that decrease the pH and therefore facilitate the solubilization of the compounds^20^. In any case, the plant growth promotion observed did not seem to be only associated to one particular activity or compound, but to the result of multiple mechanisms. Therefore, although the application of the filtrates/extracts of the studied strains caused a better plant development, none of the metabolites identified in the present study could undoubtedly be attributed as responsible of the effect, at least not without further specific experimentation. Other metabolites not identified, but also contained in the filtrates/extracts, could also have been involved.

In conclusion, the results of this study confirmed the two initial hypotheses, that the plant growth promoting activity, already observed for *F. avenaceum, S. terricola* and Xylariaceae sp. inoculated directly on plants, is caused by the metabolites that they produce, and that such metabolites can be produced in vitro and promote plant growth when applied as filtrates or extracts, to seeds or plants. The first hypothesis was evidenced by the observation of phytohormone-like substances, nutrient solubilization or antioxidant activity in the filtrates/extracts they produced, which might contain the active metabolites. The second hypothesis was confirmed by the improvement observed in most plant-growth promotion traits analysed on *Lolium multiflorum* after application of either the filtrate or extract produced in vitro to seeds or plants. Importantly, the obtaining of the filtrates/extracts was performed in vitro under standard growth conditions, but if other different conditions had been used, their metabolite profile produced or metabolite concentration might have differed. The results suggest the possibility of further technological development of these extracts, including evaluation of other different conditions to optimize their production, and of their effects in crops different from *L. multiflorum*, to determine their potential range of application, with the final objective of obtaining bio-stimulants that can improve plant yield and decrease the use of synthetic chemical products.

## Supplementary Information

Supplementary Information.

## Data Availability

The datasets generated and/or analysed during the current study are available in the Genbank repository, [www.ncbi.nlm.nih.gov; accession numbers can be found in Tables 1 and 2].
