# Supplementary material for: Plant-growth promoting activity of three fungal endophytes isolated from plants living in dehesas and their effect on Lolium multiflorum
Source: Sci Rep. 2023 May 5;13:7354. doi: 10.1038/s41598-023-34036-8 (PMC10162971; doi:10.1038/s41598-023-34036-8)
Supplement: Supplementary file 1 — Supplementary Information. [file 41598_2023_34036_MOESM1_ESM.docx]

SUPPLEMENTAL MATERIAL

Table S1. Strains of the order Xylariaceae included in the phylogenetic study. GenBank accession numbers in bold were newly generated in this study. Strain included in the present study is indicated in bold.

| **Taxa** | **Strain** | **GenBank accession number** | | | **Source** |
| --- | --- | --- | --- | --- | --- |
|  |  | **LSU** | **ITS** | ***tub2*** |  |
| *Amphirosellinia fushanensis* | HAST 91111209^HT^ |  | GU339496 | GQ495950 | [1] |
| *Amphirosellinia nigrospora* | HAST 91092308^HT^ |  | GU322457 | GQ495951 | [1] |
| *Astrocystis bambusae* | HAST 89021904 | - | GU322449 | GQ495942 | [1] |
| *Astrocystis mirabilis* | HAST 94070803 | - | GU322448 | GQ495941 | [1] |
| *Astrocystis sublimbata* | CBS 130006 | MH877041 | MH865618 | - | [2] |
| *Biscogniauxia nummularia* | MUCL 51395^ET^ | KY610427 | KY610382 | KX271241 | [3] |
| *Discoxylaria myrmecophila* | JDR 169 | - | GU322433 | GQ487710 | [1] |
| *Entoleuca mammata* | JDR 100 | - | GU300072 | GQ470230 | [1] |
| *Graphostroma platystomum* | CBS 270.87 | DQ836906 | JX658535 | HG934108 | [3, 4, 5] |
| *Hypocopra anomala* | TTI-000339 | MT903245 | - | MT901030 | [6] |
| *Hypocopra dolichopoda* | TTI-0310 | MT903247 | - | - | [6] |
| *Hypocopra rostrata* | TTI-000009 | MT903246 | MT896134 | MT901031 | [6] |
| *Kretzschmaria clavus* | JDR 114 | - | EF026126 | EF025611 | [1] |
| *Kretzschmaria guyanensis* | HAST 89062903 | - | GU300079 | GQ478214 | [1] |
| *Kretzschmaria neocaledonica* | HAST 94031003 | - | GU300078 | GQ478213 | [1] |
| *Nemania beaumontii* | HAST 405 | - | GU292819 | GQ470222 | [1] |
| *Nemania serpens* | HAST 235 | - | GU292820 | GQ470223 | [1] |
| *Podosordaria jugoyasan* | CBS 670.77 | - | AY909024 | - | [7] |
| *Podosordaria leporina* | TTI-0312 | MT903244 | - | MT901029 | [6] |
| *Podosordaria mexicana* | WSP 176 | - | GU324762 | GQ844840 | [1] |
| *Podosordaria muli* | WSP 167^HT^ | - | GU324761 | GQ844839 | [1] |
| *Podosordaria nigrobrunnea* | URM 92162^HT^ | - | MK049926 | - | [8] |
| *Podosordaria tulasnei* | CBS 128.80 | KT281897 | KT281902 | - | [9] |
| *Poronia australiensis* | MEL 2382965 | - | KP012826 | - | Bonito et al. (unp. data) |
| *Poronia erici* | DSM 107106 | - | MN954396 | - | [10] |
| *Poronia pileiformis* | WSP 88113001^ET^ | - | GU324760 | GQ502720 | [1] |
| *Poronia punctata* | CBS 656.78 | KY610496 | KT281904 | KX271281 | [3, 9] |
| *Rosellinia buxi* | JDR 99 | - | GU300070 | GQ470228 | [1] |
| *Rosellinia lamprostoma* | HAST 89112602 | - | EF026118 | EF025604 | [1] |
| *Rosellinia necatrix* | HAST 89062904 | - | EF026117 | EF025603 | [1] |
| *Sarcoxylon compunctum* | CBS 359.61 | KY610462 | KT281903 | KX271255 | [3, 9] |
| *Stromatoneurospora phoenix* | BCC 82040 | MT735133 | MT703666 | MT700438 | [6] |
| *Xylaria multiplex* | HAST 580 | - | GU300098 | GQ487705 | [1] |
| *Xylaria grammica* | BCC 20655 | MT735138 | MT703670 | - | [6] |
| *Xylaria hypoxylon* | CBS 122620^ET^ | KY610495 | KY610407 | KX271279 | [3] |
| **Xylariaceae sp.** | E051 | **OP019604** | **OK161080** | **OP856689** | Present study |

BCC: BIOTEC Culture Collection, National Center for Genetic Engineering and Biotechnology (BIOTEC), Khlong Luang, Pathumthani, Thailand; CBS: Westerdijk Fungal Biodiversity Institute, Utrecht, the Netherlands; DSM: Leibniz Institute DSMZ - German Collection of Microorganisms and Cell Cultures GmbH, Braunschweig, Germany; HAST: Herbarium, Research Center for Biodiversity, Academia Sinica, Taipei, Taiwan; MEL: National Herbarium of Victoria, Melbourne, Australia; MUCL: Mycothèque de l'Université catholique de Louvain, Louvain-la-Neuve, Belgium; TTI: Texas Therapeutics Institute Collection, Houston, Texas, USA; URM: University Recife Mycologia Collection, Universidade Federal de Pernambuco, Recife, Brazil; WSP: Charles Gardner Shaw Mycological Herbarium, Washington State University, Pullman, Washington, USA; JDR: personal collection of Jack D. Rogers; ET and HT indicate ex-epitype and holotype strains, respectively.

References:

[1] Hsieh, H-M., Lin, C-R., Fang, M-J., Rogers, J. D., Fournier, J., Lechat, C., Ju, Y-M. Phylogenetic status of Xylaria subgenus Pseudoxylaria among taxa of the subfamily Xylarioideae (Xylariaceae) and phylogeny of the taxa involved in the subfamily. *Mol. Phylogenet. Evol.* **54(3)**, 957-969 (2010).

[2] Vu, D., Groenewald, M., De Vries, M., Gehrmann, T., Stielow, B., Eberhardt, U., Al-Hatmi, A., Groenewald, J. Z., Cardinali, G., Houbrake, J., Boekhout, T., Crous, P. W., Robert, V., Verkley, G. J. M. Large-scale generation and analysis of filamentous fungal DNA barcodes boosts coverage for kingdom fungi and reveals thresholds for fungal species and higher taxon delimitation. *Stud. Mycol*. **92**, 135–154 (2019).

[3] Wendt, L., Sir, E.B., Kuhnert, E. Heitkämper, S., Lambert, C., Hladki, A. I., Romero, A. I., Luangsa-ard, J., Srikitikulchai, P., Peršoh, D., Stadler, M. Resurrection and emendation of the Hypoxylaceae, recognised from a multigene phylogeny of the Xylariales. *Mycol. Progress* **17**, 115–154 (2018).

[4] Zhang, N., Castlebury, L. A., Miller, A. N., Huhndorf, S. M., Schoch, C. L., Seifert, K. A., Rossman, A. Y., Rogers, J. D., Kohlmeyer, J., Volkmann-Kohlmeyer, B., Sung, G-H. An overview of the systematics of the Sordariomycetes based on a four-gene phylogeny. *Mycologia,* **98**, 1076-1087 (2006).

[5] Stadler, M., Læssøe, T., Fournier, J., Decock, C., Schmieschek, B., Tichy, H-V., Peršoh, D. A polyphasic taxonomy of Daldinia (Xylariaceae). *Stud. Mycol.* **77**, 1-143 (2014).

[6] Becker, K., Wongkanoun, S., Wessel, A., Bills, G. F., Stadler, M., Luangsa-ard, J. J. Phylogenetic and chemotaxonomic studies confirm the affinities of *Stromatoneurospora phoenix* to the coprophilous Xylariaceae*. J. Fungi* **6**, 144 (2020).

[7] Peláez, F., González, V., Platas, G., Sánchez Ballesteros, J., Rubio, V. Molecular phylogenetic studies within the Xylariaceae based on ribosomal DNA sequence. *Fungal Divers*. **31**, 111-134 (2008).

[8] Crous PW, Carnegie AJ, Wingfield MJ, et al. Fungal Planet description sheets: 868-950. *Persoonia*. **42**:291-473 (2019).

[9] Senanayake, I.C., Maharachchikumbura, S.S.N., Hyde, K.D. et al. Towards unravelling relationships in Xylariomycetidae (Sordariomycetes). *Fungal Divers*. **73**, 73–144 (2015).

[10] Perić, B., Wendt, L. Première récolte monténégrine d’une espèce menacée et protégée en Europe: *Poronia erici* (Xylariaceae). *Ascomycete.org*, **9 (7)**, 275-289 (2017).

Table S2. Summary of the one-way ANOVAs showing the effect of the fungal filtrate on each parameter evaluated for both the seed germination and the seedling growth assays. DF, degree of freedom; *F* values, and *P*-values.

| **Seed assay** | shoot length (cm) | Root length (cm) | Plant dry weight (mg) | Number of roots | Vigour index |
| --- | --- | --- | --- | --- | --- |
| DF | 4 | 4 | 4 | 4 | 4 |
| *F value* | 86.04 | 0.41 | 6.24 | 16.1 | 122.33 |
| *P-value* | <0.001 | 0.798 | 0.002 | <0.001 | <0.001 |
| **Seedling assay** | Shoot length  (cm) | Root length  (cm) | Plant dry weight  (mg) | Number of roots | Chlorophyll (SPAD units) |
| DF | 4 | 4 | 4 | 4 | 4 |
| *F value* | 24.73 | 15.44 | 129.43 | 4.97 | 9.04 |
| *P-value* | <0.001 | <0.001 | <0.001 | 0.006 | 0.002 |

Table S3. Summary of the one-way ANOVAs showing the effect of the fungal filtrate on plant growth parameters in *Lolium multiflorum* plants under greenhouse conditions. DF, degree of freedom; *F* values, and *P*-values.

|  | Shoot length (cm) | Root length (cm) | Shoot dry weight (mg) | Root dry weight (mg) | Plant dry weight (mg) | Number of roots | Number of tillers | Chlorophyll (SPAD units) |
| --- | --- | --- | --- | --- | --- | --- | --- | --- |
| DF | 3 | 3 | 3 | 3 | 3 | 3 | 3 | 3 |
| *F value* | 8.55 | 4.98 | 14.37 | 21.57 | 18.96 | 4.00 | 9.03 | 1.49 |
| *P-value* | 0.001 | 0.013 | <0.001 | <0.001 | <0.001 | 0.027 | 0.001 | 0.254 |

Figure S1. Mean relative humidity (HR) and mean maximum (Tmax) and minimum (Tmin) temperatures during the greenhouse experiments in 2019
